# A Five-Year Data Report of Long-Term Central Venous Catheters Focusing on Early Complications

**DOI:** 10.1155/2019/6769506

**Published:** 2019-12-10

**Authors:** Harald Lenz, Kirsti Myre, Tomas Draegni, Elizabeth Dorph

**Affiliations:** ^1^Oslo University Hospital, Division of Emergencies and Critical Care, Department of Anaesthesiology, Postbox 4950 Nydalen, 0424 Oslo, Norway; ^2^Oslo University Hospital, Division of Emergencies and Critical Care, Department of Research and Development, Postbox 4950 Nydalen, 0424 Oslo, Norway

## Abstract

**Background:**

Long-term venous access has become the standard practice for the administration of chemotherapy, fluid therapy, antibiotics, and parenteral nutrition. The most commonly used methods are percutaneous puncture of the subclavian and internal jugular veins using the Seldinger technique or surgical cutdown of the cephalic vein.

**Methods:**

This study is based on a quality registry including all long-term central venous catheter insertion procedures performed in patients >18 years at our department during a five-year period. The following data were registered: demographic data, main diagnosis and indications for the procedure, preoperative blood samples, type of catheter, the venous access used, and the procedure time. In addition, procedural and early postoperative complications were registered: unsuccessful procedures, malpositioned catheters, pneumothorax, hematoma complications, infections, nerve injuries, and wound ruptures. The Seldinger technique using anatomical landmarks at the left subclavian vein was the preferred access. Fluoroscopy was not used.

**Results:**

One thousand one hundred and one procedures were performed. In eight (0.7%) cases, the insertion of a catheter was not possible, 23 (2.1%) catheters were incorrectly positioned, twelve (1.1%) patients developed pneumothorax, nine (0.8%) developed hematoma, and three (0.27%) developed infection postoperatively. One (0.1%) patient suffered nerve injury, which totally recovered. No wound ruptures were observed.

**Conclusions:**

We have a high success rate of first-attempt insertions compared with other published data, as well as an acceptable and low rate of pneumothorax, hematoma, and infections. However, the number of malpositioned catheters was relatively high. This could probably have been avoided with routine use of fluoroscopy during the procedure.

## 1. Introduction

The use of tunneled long-term venous catheters has become the standard practice for the administration of chemotherapy, fluid therapy, antibiotics, and parenteral nutrition. Long-term venous access was first described by Broviac et al. [1] and Hickman et al. [2] in the 1970s, and in 1982, Niederhuber et al. [3] introduced a totally implanted venous port system.

There are several approaches to implantation; percutaneous puncture using the Seldinger technique [4] or surgical cutdown [5] are the most frequent methods. For surgical cutdown, the cephalic vein is the most commonly used vein [6] and requires a venotomy to be performed to allow catheter insertion. In the case of percutaneous puncture, the subclavian and internal jugular veins are the most commonly used [7]. 

The most common complications related to the insertion of venous ports/central venous catheters (CVC) are malposition, pneumothorax, accidental arterial puncture, hematoma, infection, nerve injury, wound rupture, malignant arrhythmias, and thrombosis [8, 9].

Our study is based on a quality registry of percutaneous tunneled long-term central venous catheterizations performed at our department during a five-year period. The aim of the study was to investigate procedural and early postoperative complications.

## 2. Methods

This study is based on a quality registry containing data on all tunneled long-term central venous catheterization procedures performed at the Department of Anaesthesiology, Oslo University Hospital, Ullevaal in the period lasting from 01.01.2012 to 31.12.2016. The data for the first three years are retrospective, and for the two remaining years, the data were prospectively collected. Both the creation of the quality registry and the publishing of the registry data have been approved by the hospital's data protection officer, and the registry data are stored and processed on a local data server approved by the data protection officer.

In the quality registry, the patient data were registered and analyzed for procedural and early postoperative complications. The following demographic data were registered: sex, age (yr.), weight, height, and body mass index (BMI). Only patients >18 years were included.

In addition, the patients' main diagnosis and indications for the procedure were registered. For one single patient, several indications could be registered.

The following blood tests were routinely performed and registered in most of the patients: hemoglobin, activated partial thromboplastin time (APTT), international normalized ratio (INR), and platelets.

The identity of the operator (an anaesthesiologist) and the assistant (a nurse anaesthetist) was also registered, as well as the type of catheter and the venous access. Three types of catheters were used: Braun Celcite® Implantable Venous Port (single or double lumen), Bard Hickman® Catheter (single, double, or triple lumen), and Bard Hemostar® Long-Term Hemodialysis Catheter.

The preferred site of access was the left subclavian vein, except for in patients with previous surgery for left-sided breast cancer and in patients with a preference for the right side. In cases where access via the subclavian vein was not possible, the procedure was performed using an access via the jugular vein at the same side. When access via the jugular vein was also unsuccessful, the procedure was defined as unsuccessful. Fluoroscopy was not used during the procedures, and when the procedures failed, a new procedure in cooperation with a radiologist using ultrasound and fluoroscopy was usually performed the following day.

Occasionally, the preferred site of access had to be changed in some patients due to prior central vein thrombosis.

The procedures were mainly performed by four experienced anaesthesiologists (>10 years as consultants) using the Seldinger technique [4]. However, some procedures were also performed by less experienced anaesthesiologists (in total 13). For the detection and puncture of the subclavian vein, ultrasound-guided technique was not used, only anatomical landmarks. However, to obtain access via the jugular vein, ultrasound guidance was used in most cases. Lidocaine 10 mg/ml with adrenalin 5 *μ*g/ml was administered as local anaesthesia, and for sedation, a combination of midazolam and fentanyl was given. In some patients, propofol was additionally administered. In patients experiencing pain during the last part of the procedure, alfentanil was administered. Prophylactic antibiotics were not used. Patients with platelet levels less than 20–30 × 10/L received platelet infusion immediately before or during the procedure. The catheters were locked with heparin 100 IE/ml at the end of the procedure. The procedure time and total operating room time were registered. In all cases, a chest X-ray was performed two hours or more after the catheter insertion.

The following main complications were registered for the whole five-year period: unsuccessful procedure, malpositioned catheters, pneumothorax, with or without the need of chest tube drainage, hematoma, infection (local or systemic the first seven days following insertion), nerve injury, wound rupture, and malignant arrhythmias during the procedure. We assumed that an infection which occurred the first week indicated a procedure-related infection. Infections occurring later than one week after insertion were not registered. Accidental arterial punctures were registered only for the two last prospective years and is therefore not included in the results.

Two different groups of anaesthesiologists performing the insertions were also compared: the experienced group (the four anaesthesiologists who inserted most of the catheters) and the less experienced group (the other 13). Our hypothesis was that the experienced group had fewer complications and used less time performing the procedures. We also investigated if there were any significant differences regarding complication rates between the operators at an individual level. The data were analyzed using the independent samples *t*-test for continuous variables with nearly normal distribution and nonparametric test for not normal distributed variables. The chi-squared test was used for categorical data. All comparison tests were two-tailed. The Statistical Package for the Social Sciences (SPSS) version 23 (SPSS Inc., Chicago, IL) was used for analyzing the data. The significance level was set to 0.05.

## 3. Results

During the five-year period, a total of 1101 procedures were performed, and 1099 tunneled long-term catheters were inserted. Two of the patients received a short-term central venous catheter. One patient had five catheters inserted, one patient had four, 13 patients had three, and 56 patients had two catheters inserted during the five-year period.

The demographic data are presented in Table 1, and blood sample results are presented in Table 2. The demographic data for the three retrospective years were compared with the data for the two prospective years in order to see if the patient populations had changed. We did not find any statistical differences between these data (data not shown).

The five most common indications for the procedure were chemotherapy (77.7%), difficult vein access (37.0%), parenteral nutrition (15.3%), administration of other medications (8.6%), and fluid treatment (4.9%). More than one indication could be registered for one single patient.

Four consultants performed 76% (*n* = 837) of the tunneled catheter insertion procedures. In total, 17 anaesthesiologists, both consultants and residents, performed the procedures.  Table 3 shows the catheter inserted and the vein used, and the main complications are presented in Table 4. In the comparison of the complication rates between the retrospective and prospective data, we did not find any differences (data not shown).

The mean procedure time was 41 min (range 15–210 min). The mean operating room time was 71 min (range 35–265 min). For sedation, four different drugs were used: fentanyl (mean 0.1 mg, range 0–0.45 mg), midazolam (mean 3.0 mg, range 0–10 mg), alfentanil (mean 0.06 mg, range 0–2.0 mg), and propofol (mean 26.6 mg, range 0–1050 mg).

In the comparison of the experienced group (the four consultants performing 76% of the procedures) to the other 13 anaesthesiologists, we found that there were statistically significant differences in the procedure time and the operating room time in favor of the experienced group (procedure time: mean 38 min vs. 49 min—*p* < 0.001; operating room time: mean 67 min vs. 83 min—*p* < 0.001). There were no significant differences in the number of unsuccessful procedures, malpositioned catheters, and in the rate of pneumothorax, hematoma, infection, nerve injury, and wound rupture between the two groups. In addition, we did not find any significant differences regarding the complication rates between the individual operators (data not shown).

## 4. Discussion

In our study, we found a high success rate of first-attempt insertions, as well as an acceptable and low rate of pneumothorax, hematoma, and infections. However, the number of malpositioned catheters was relatively high.

It has not yet been established which of the techniques, Seldinger or surgical cutdown, provide the best results [10, 11].

A Cochrane review from 2016 showed a greater primary success rate of totally implantable venous access port (TIVAP) placements with the Seldinger technique (both the jugular and the subclavian vein) compared with the venous cutdown technique via the cephalic vein [10]. This review showed no differences in overall perioperative and postoperative complication rates, but that the use of the Seldinger technique via the subclavian vein causes a higher overall risk of catheter-related complications.

A systematic review and meta-analysis by Orci et al. demonstrated a higher primary success rate with percutaneous subclavian vein puncture vs. surgical venous cutdown. In this review, no significant differences in terms of risk of pneumothorax, hematoma, or infectious events were found; however, pneumothorax only occurred after the percutaneous approach [12].

Of the 1,101 procedures in our material, 1,093 (99.3%) of the long-term catheters were successfully implanted at the first attempt. Compared with other published data, this is a high success rate [10, 13, 14]. In these studies, the primary success rate was between 84% and 95%. In two of these studies, the first attempt was defined as failure of the primary approach [13, 14].

We defined the success rate after also performing via the jugular vein at the same side when access via the subclavian vein failed.

The usual reasons for an unsuccessful surgical cutdown procedure via the cephalic vein are that the vein is impossible to locate, too small, or occluded [13]. When missing the first attempt in that study, they converted to another access resulting in a success rate of 97.4%. The reasons for unsuccessful procedures via the subclavian vein are inability to advance the wire/catheter or failure to puncture the subclavian vein, [13, 14] which is also the case for unsuccessful procedures in our study (Table 4). In two of our eight unsuccessful procedures, two patients had a short-term central venous catheter inserted during the same procedure. In the six other cases, long-term catheters were successfully placed in a fluoroscopy lab, usually the day following the first procedure, and often in cooperation with a radiologist (Table 4).

A recent Cochrane systematic review summarizes the current evidence for ultrasound (US) guidance vs. anatomical landmark techniques for central venous catheter (CVC) placement in the subclavian vein with regard to complications [15]. The meta-analysis showed that the use of ultrasound resulted in a reduced rate of accidental arterial punctures (US, 2/242 (0.8%)) vs. landmark (15/256 (5.9%)) and hematoma (US, 3/242 (1.2%)) vs. landmark (17/256 (6.6%)).

Although the use of ultrasound offers small gains in safety and quality, the authors concluded that the meta-analysis does not generally support the use of ultrasound for CVC placement in the subclavian vein.

Ultrasound guidance can improve patient safety and procedural quality during CVC placement in the subclavian vein. Some medical societies recommend the use of ultrasound for CVC placement also in the subclavian vein, even though the evidence is debatable [16]. Data from survey studies show that there is still a gap between the existing evidence and guidelines and the use of ultrasound in clinical practice [17]. It is important to keep in mind that it is more technically challenging to use ultrasound for CVC placement in the subclavian vein than in the internal jugular vein. Providing adequate training for all operators in a department can also be demanding. In addition, purchasing and maintaining ultrasound machines is expensive [18].

The data in our registry demonstrated that the anatomical landmark technique for subclavian vein access has a complication rate comparable to that of the ultrasound technique [14, 15].

Interestingly, a study by Ertel et al. indicates that the outcome depends more on the operator than on the technique per se [19]. In their study, they found that individual surgeons were the strongest predictors of increased operating room time, likelihood of switching to an alternative method, and procedural complications. The only significant difference we found was that the experienced group in our study spent less time performing the procedure. Regarding complications, we did not find any significant differences between the two groups or at an individual operator level. The sample size is probably too small to find any differences because the complication incidence was low.

In our study, there were 23 (2.1%) malpositioned catheters, 22 were repositioned in a fluoroscopy lab the next day (Table 4), and one catheter was left unchanged.

We did not use fluoroscopy during the primary procedures. In many published studies, fluoroscopy is the standard [11, 14, 20, 21]. The need of a second procedure due to malpositioning is time consuming, inconvenient for the patient, and in some cases leads to delayed initiation of therapy. A second procedure might also increase the risk of infection, even though none of our 23 patients developed infections. In our opinion, 2.1% malpositioned catheters is a high number. We therefore believe that fluoroscopy should be considered. As an alternative to fluoroscopy, an ultrasound-guided supraclavicular approach to confirm the guidewire position and the final CVC tip position after insertion is a possibility to be considered [22, 23].

Twelve (1.1%) of the patients developed pneumothorax (PT) (Table 4), and four of them did not need a chest tube. Three procedures were reported as uncomplicated, whereas in nine of the procedures, the anaesthesiologist reported several attempts, arterial puncture, or air in the syringe. Low body mass index (BMI) seems to be a risk factor for pneumothorax; mean BMI in the PT group was 20.4, and 24.5 (*p*=0.01) in the rest of the patients. This is in line with earlier findings [24].

A multicenter trial comparing three different anatomical sites (subclavian, jugular, or femoral vein) for nontunneled central venous catheterization in patients in the adult intensive care unit [25] demonstrated pneumothorax in 1.5% of the cases when the CVC was placed in the subclavian vein. In that study, both techniques (anatomical landmarks and ultrasound) were used in all three veins accesses.

Compared to published studies on insertion of a Port-A-Cath, our pneumothorax rate is acceptable. The lowest rate that we have found in the literature has been documented by Tsai et al. [7]. In their study, one surgeon inserted 1848 Port-A-Caths using the Seldinger technique and anatomical landmarks, and only 0.3% of the patients developed pneumothorax. All patients in their study had a chest X-ray taken within 30 min of the procedure, which may have been too early to detect all cases of pneumothorax, especially nonsymptomatic cases. Mudan et al. [14] used ultrasound guidance to puncture the subclavian vein under the clavicular, and twelve of 1000 patients (1.2%) in their study developed pneumothorax.

Nine (0.8%) of the patients in our study developed local hematoma postoperatively. However, none of the catheters were removed, and all could be used. This result is acceptable compared to other studies where bleeding associated with CVC insertion has a reported incidence of 0.5–1.6% [26]. Of these nine patients in our study, five used dalteparin or acetylsalicylic acid, two of these five patients had in addition prolonged APTT. Two patients had pathological lab data (prolonged APTT or low platelet count). In one patient, two arterial punctures and two vein punctures were performed during the same procedure. One procedure was described as uncomplicated with normal lab results and no use of anticoagulation. Pathological lab data and the use of anticoagulants and platelet inhibitors were known before the procedures. The risk of discontinuing the anticoagulation therapy prior to the procedures was considered as more hazardous than the risk of bleeding, as these patients often had a history of thromboembolism.

In three procedures in our study described as uncomplicated, postoperative wound infections were detected. All three were women suffering from cancer. One of the patients had diabetes, and two had a low leucocyte level (1.8 and 2.8 × 10^9^/L). Two patients had fever 2-3 days after insertion and positive blood cultures (one *Staphylococcus aureus* and one *Staphylococcus lugdunesis*). One patient had no fever, but local rubor and pain, and microbiological tests of the wound secretions detected Staphylococcus epidermidis. All three catheters were removed, and the patients were treated with antibiotics. All recovered. Mudan et al. [14] did not experience any infections in the immediate postoperative period (not defined); the patients received one single prophylactic dose of antibiotic (amoxicillin 1.2 g). In two other studies, no prophylactic antibiotic therapy was given, [27, 28] but the reported infection rates are difficult to compare to our data, as the observation time is different. We believe that an infection rate of three out of 1101 is acceptable, and that antibiotic prophylaxis is not necessary. In general, prophylactic antibiotics are not routinely recommended for CVC insertion [29–31].

In our material, there was only one report of nerve injury, with symptoms that recovered totally. However, as three years of our data are retrospective, we may have missed some injuries; symptoms of a minor degree compared to the patients' serious disease may not have been reported.

There were no wound ruptures. Even though three years of the data are retrospective, wound ruptures would have been reported as a primary complication after the procedure.

## 5. Conclusion

This study is based on a single institution registry of tunneled long-term central venous catheterizations covering a five-year period. Compared with other studies, we have an acceptable and low rate of pneumothorax, hematoma, and infections. We have a high success rate of first-attempt insertions compared with other published data. Even though our group used the anatomical landmarks technique at the subclavian vein, the complication rate is comparable to the use of ultrasound technique. To achieve these results, the procedure should be performed by a limited number of operators, and the total number of catheters inserted needs to be large enough to ensure that each operator inserts catheters on a regular basis. The high occurrence of malpositioned catheters is a cause of concern, and we will therefore consider using fluoroscopy in our future procedures.

## Figures and Tables

**Table 1:** Demographic data.

Total number *N* = 1101	Median (25^th^–75^th^ percentiles) and number/percent	Min/max and number/percent
Age (yr.)	59.0 (49–67)	18–86
Weight (kg)	70.0 (60–82)	24–60
Height (cm)	171.0 (165–178)	137–205
BMI	23.8 (20.7–27.5)	10.7–47.3
Sex (women/men)	624/57%	477/43%

The values are median (25^th^–75^th^ percentiles) or number/percent. BMI = body mass index; kg/m [2].

**Table 2:** Blood sample tests.

Test *N* = 1101	Number of tests and (missing)	Mean (±SD)	Min/max
Hgb	1088 (13)	11.7 (±1.68)	7.2–18.1
APTT	940 (161)	35.3 (±5.4)	3–80
INR	1022 (79)	1.05 (±0.13)	0.8–1.9
TC	1077 (24)	288 (±172)	4–3232

Hgb = hemoglobin, APTT = activated partial thromboplastin Time, INR = international normalized ratio, TC = thrombocytes. The values are indicated as mean ± SD or minimum/maximum values.

**Table 3:** Type of catheter and vein access.

Catheters and vein access	Number (*N* = 1101)	The experienced group (4 anaesthesiologists)	The less experienced group (13 anaesthesiologists)
Long-term implantable venous port (single or double lumen)	999 (998/1)		
Hickman (single, double, triple lumen, or not specified)	74 (40/17/1/16)		
Long-term hemodialysis catheter	26		
Short-term CVC	2		
Left subclavian vein	870	659 (75.7%)	211 (24.3%)
Right subclavian vein	190	144 (75.8%)	46 (24.2%)
Right jugular vein	28	26 (92.9%)	2 (7.1%)
Left jugular vein	13	9 (69.2%)	4 (30.7%)

CVC = Central venous catheter. The values are number and percent.

**Table 4:** Main complications.

Total number *N* = 1101	Number and percent	Details
Unsuccessful first procedure	8 (0.7%)	Two patients: right subclavian vein: narrow between the collar bone and the first costa. Not possible to insert the long-term catheter. A smaller short-term CVC was successfully introduced into the vein
		One patient: history of several long-term catheters. Access through the right subclavian and then the right internal jugular (IJ) vein. The guidewire stopped inside the veins. Next day through fluoroscopy: recanalization through a thrombotic brachiocephalic vein
		Two patients with several attempts via the left subclavian and left IJ vein: blood response, but impossible to enter the vein with the guidewire. One of the patients had a history of several long-term catheters. This patient was diagnosed with a central thrombus in the left brachiocephalic vein. The following day, the two patients had uncomplicated access through the right subclavian vein. One patient with the same problem, but on the opposite side
		One patient with a history of several long-term catheters: Access through the left subclavian vein. Not possible to introduce the catheter. Later contrast-enhanced fluoroscopy revealed a narrow left brachiocephalic vein. A Hickman catheter was inserted without complications in the right subclavian vein
		One patient with extreme obesity: left subclavian and left IJ vein. Several attempts. not possible to enter with the guidewire. The artery was also punctured. Successfully inserted long-term catheter via the right IJ vein the following day in a fluoroscopy lab

Malposition	23 (2.1%)	Six left subclavian vein to right subclavian vein and five left subclavian vein to left IJ vein
		One right subclavian vein to left subclavian vein and six right subclavian vein to right IJ vein
		One right subclavian vein to left brachiocephalic vein and two left subclavian vein to right brachiocephalic vein
		One right subclavian vein to right atrium and one left subclavian vein to left brachiocephalic vein
		One catheter was left unchanged. Nine catheters were repositioned using a snare via a femoral vein access. Thirteen catheters were repositioned by opening the section under the collar bone and manipulating the catheter in the right position using fluoroscopy

Pneumothorax (PT)	12 (1.1%)	Eight needed a chest tube, and four did not need a chest tube
		Three procedures were described as uncomplicated. Nine were described with several attempts, arterial puncture or air in the syringe. Mean BMI in the PT group: 20.4. Mean BMI in the non-PT group: 24.5. *P* = 0.01

Bleeding (hematoma)	9 (0.8%)	No catheters were removed. Five patients used dalteparin or acetylsalicylic acid, and two of them had prolonged APTT in addition. Two patients had pathological lab (prolonged APTT or low platelet level). One patient had two arterial punctures and two vein punctures. One procedure was uncomplicated with normal lab and no use of anticoagulation

Postoperative infection	3 (0.27%)	The procedures were described as uncomplicated. All three patients were women with cancer. One patient had diabetes. Two of the patients had low leucocyte levels (1.8 and 2.8 × 10^9^/L). Two patients had fever 2-3 days after insertion and positive blood cultures (staphylococcus in both). One patient had no fever, but local rubor and pain. Staphylococcus was found in the wound secretions. All three catheters were removed, and the patients recovered

Nerve injury	1 (0.1%)	One patient had short-lasting pain in the arm, which totally recovered
Wound rupture	0 (0%)	
Malign arrhythmias	0 (0%)	Malignant arrhythmias are defined as arrhythmias requiring intervention with drugs or cardioversion

Values are number/percent. BMI = body mass index; kg/m [2]. IJ vein = internal jugular vein. CVC = central venous catheter.

## Data Availability

The quality register data are stored on a server which is governed by Oslo University Hospital. As the patient data are interpreted as sensitive, we are unfortunately not allowed by the hospital's lawyers to release the data.
